# TRPC3 as a Target of Novel Therapeutic Interventions

**DOI:** 10.3390/cells7070083

**Published:** 2018-07-22

**Authors:** Oleksandra Tiapko, Klaus Groschner

**Affiliations:** Gottfried-Schatz-Research-Center—Biophysics, Medical University of Graz, Neue Stiftingtalstrasse 6/D04, 8010 Graz, Austria; oleksandra.tiapko@medunigraz.at

**Keywords:** transient receptor potential channels, TRPC3 pharmacology, channel structure, lipid mediators, photochromic ligands

## Abstract

TRPC3 is one of the classical members of the mammalian transient receptor potential (TRP) superfamily of ion channels. TRPC3 is a molecule with intriguing sensory features including the direct recognition of and activation by diacylglycerols (DAG). Although TRPC3 channels are ubiquitously expressed, they appear to control functions of the cardiovascular system and the brain in a highly specific manner. Moreover, a role of TRPC3 in immunity, cancer, and tissue remodeling has been proposed, generating much interest in TRPC3 as a target for pharmacological intervention. Advances in the understanding of molecular architecture and structure-function relations of TRPC3 have been the foundations for novel therapeutic approaches, such as photopharmacology and optochemical genetics of TRPC3. This review provides an account of advances in therapeutic targeting of TRPC3 channels.

## 1. Introduction to TRPC3

Mammalian transient receptor potential (TRP) channels of the classical subfamily (TRPC) are closely related to the founding member dTRP, which was discovered as a critical element in *Drosophila* visual transduction [1]. In human tissues, TRPCs typically serve signal transduction pathways downstream of G protein-coupled receptors [2]. All TRPCs are controlled by and able to sense membrane lipids [3,4], where TRPC3/6/7 channels display a direct mechanism of activation via diacylglycerols [5,6], which is generated in response to receptor-phospholipase C pathways. Like all other TRPC channels, TRPC3 features six transmembrane spanning segments with nitrogen (N) and carbon (C) termini residing in the cytoplasm. TRPC3 assembles into tetrameric complexes in which the cytoplasmic termini interact to form an inverted bell-shaped cytoplasmic layer, as revealed by single-particle cryo-electron microscopy (cryo-EM) [7,8]. The tetrameric assembly constitutes a cation permeation path with a selectivity filter harboring negatively charged residues (E630 in the 848aa variant; isoform 3/Q13507-3 in UniProt) to determine calcium ion (Ca^2+^) transport within the pore domains, connecting transmembrane domains 5 and 6 (TM5 and TM6) [7,9]. Multiple cytoplasmic regulatory domains have been identified, including a highly conserved proline-rich and calmodulin/IP_3_ receptor binding (CIRB) region in the C-terminus [10,11], which enable the channel to serve multimodal signaling functions. Initially, the channel was implicated in store-operated Ca^2+^ entry processes [12,13] but later on, a consent was reached among researchers that the prominent mechanism of TRPC3 activation and TRPC3-mediated Ca^2+^ signaling is based on a direct interaction with diacylglycerol. This was found to occur within a lateral gating fenestration of the pore domain [14]. Like its DAG-sensitive relatives TRPC6 and TRPC7, TRPC3 has been implicated in a wide array of pathologies and disorders ranging from tumors to cardiac arrhythmias [15]. Notably, expression of TRPC3 varies among tissues and their developmental state as well as cell phenotype. A prominent functional role of TRPC3 has been detected in both proliferating cells, such a vascular progenitors [16,17], but also in differentiated cell types [18,19]. Overall, pharmacological targeting of TRPC3 with high specificity and spatiotemporal precision has become feasible and emerged as an attractive perspective in TRPC pharmacology.

## 2. Potential Role of TRPC3 in Human Disease

Ca^2+^ influx is an essential determinant of cell function and fate, and TRPC3 serves to regulate Ca^2+^ entry via its nonselective permeation pathway by multiple mechanisms, including functional interaction with the sodium-calcium exchanger NCX1 [20,21,22,23]. TRPC3 mRNA was detected in both excitable and non-excitable cells, and changes in expression levels are reportedly correlated with pathological processes and organ disorders [24]. A gain in TRPC3 function was found to be associated with pathologies of the cardiovascular system and brain. In the heart, TRPC3 channels were confirmed as a major target of the angiotensin II- and noradrenaline-induced nuclear factor of activated T cells (NFAT) activation involved in maladaptive cardiac remodeling and arrhythmias [20,22,25,26]. TRPC3 overexpression and/or gain-of-function depolarizes myocytes, promotes the calcineurin/NFAT pathway, is involved in adverse mechanical stress responses, hypertrophy, and heart failure [25,26]. Importantly, NFAT signaling in myocytes has been linked to direct Ca^2+^ entry via TRPC3 channels [27]. Nonetheless, the pathophysiologicial role of TRPC3 in the heart appears, for a large part, to be based on its expression and function in cardiac fibroblasts. TRPC3 was identified as a crucial player in the proliferation and differentiation of fibroblasts in the myocardium and its activity was found to promote fibrosis, structural remodeling and arrhythmias, specifically atrial fibrillation [28,29,30].

TRPC3 channels are also expressed in other cardiovascular cells, including vascular smooth muscle and endothelial cells [31,32,33]. TRPC3 has been proposed to govern both the fate of endothelial progenitor cells and functions in the mature endothelium specifically vasodilatory responses [16]. TRPC3-mediated Ca^2+^ was reported to trigger NO-mediated [34] and NO-independent vasodilation [33]. For vascular smooth muscle, Dietrich et al. showed that up-regulated expression of TRPC3 channels, which features constitutive activity, is associated with high blood pressure in TRPC6-deficient mice [35]. Similar to cardiac muscle, a role of TRPC3 in phenotype transitions and vascular remodeling was suggested [36].

TRPC3 expression is detectable throughout the brain with prominent levels in cerebellar Purkinje cells in the adult mouse brain [37]. Notably, up-regulation of the neuronal TRPC3 conductance by the gain-of-function mutation T635A (moonwalker; *Mwk*) was shown to lead to Ca^2+^-dependent degradation of Purkinje cells and, as a consequence, to impaired motor coordination [38,39]. In the hippocampus, TRPC3 activity was found to be negatively correlated with contextual fear memory [40].

A role in non-excitable cell signaling was proposed for the immune system. TRPC3 was reported to control Ca^2+^ waves and to facilitate the response to antigen stimulation [41]. Phillip et al. detected defects in the TRPC3 gene of immune cell lines with impaired Ca^2+^ signaling, which were initially described by Fanger et al. [42]. Phillip et al. were able to restore the Ca^2+^ influx and activation of T-cells by overexpression of functional TRPC3 channels [43]. Hence, TRPC3 was suggested to contribute to Ca^2+^ signaling in immune cells alongside the prominent players stromal interaction molecule (STIM) and Orai, which constitute the classical calcium release-activated Ca^2+^ channel (CRAC) conductance [44].

Growing consensus states that TRPC molecules impact on nearly all “cancer hallmarks” and drive cancer progression [45]. In particular, TRPC3 was found as an ion channel that governs proliferation and migration of a variety of tumor cells, including melanoma [46], lung [47], bladder [48], ovarian [49], and breast [50] cancers.

Current knowledge on the role of TRPC3 in most investigated pathologies suggests that channel blockers might be suitable for disease management. This has been suggested for cardiac fibrosis and hypertrophy [29,51], coronary stenosis [36], and melanoma [46]. Nonetheless, for certain disorders, selective block of TRPC3 channel functioning might not be a useful therapeutic strategy since the protein’s cellular role is more complex, and TRPC3 function has also been assigned to beneficial effects, such as stabilization of cardiac contractility, and excitability and vasodilation or promotion of immune responses. Not only should the expression levels and overall channel activity be considered, but also the cell-type specific signaling signature of TRPC3 channels, which depends on factors like subcellular localization, composition of pore complex, and input signaling pattern, and are likely of relevance for disease etiology. The ability of TRPC proteins to assemble into specific heteromeric complexes, for which stoichiometry is likely to determine signaling features as well as sensitivity to pharmacological intervention, has long been recognized [52,53,54]. Moreover, native TRPC channels have been shown to operate in cell-specifically organized signalplexes, which enable efficient interactions with downstream signaling elements, such as CaN [27] or the electrogenic Ca^2+^ signaling partner NCX1 [22]. Dynamic organization of TRPC3 into such cell-type specific signalplexes, along with the TRPC channels cycling between activated, inactivated, and desensitized states, needs consideration as a basis of cell-type specific signaling and therapeutic targeting of TRPC3. In this context, more refined pharmacological interventions including also channel activators and modulators might be of value for therapeutic applications.

## 3. Pharmacological Inhibitors

**Figure 1:**
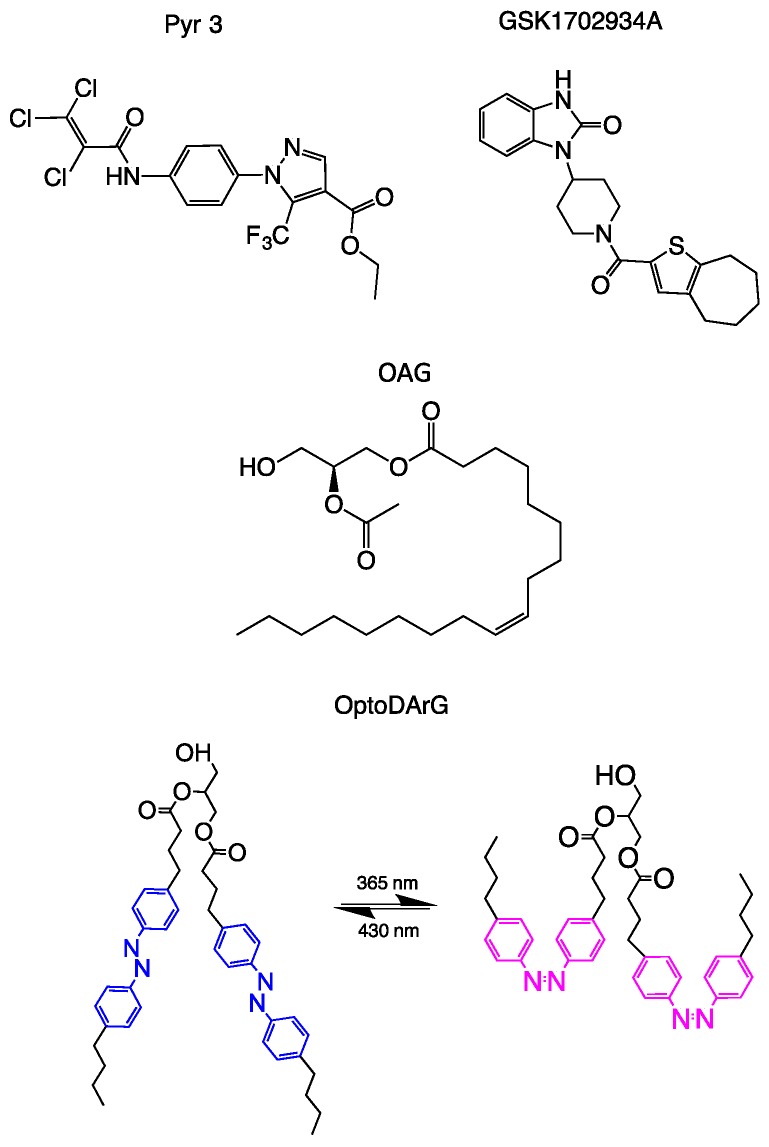
Chemical structures of prototypical antagonist and agonists of transient receptor potential channel 3/6 (TRPC3/6): Pyrazole 3 (Pyr3) as a most commonly used pore blocker; GSK1702934A and 2-Acetyl-1-oleoyl-sn-glycerol (OAG) represent channel agonists (a synthetic, non-lipid activator, and a diacylglycerol/lipid, respectively); OptoDArG as a photochromic agonist (photoswitchable lipid) and a powerful tool for precise control of TRP channels (TRPC) activity.

Early attempts to identify and characterize TRPC3 channel function were based on non-specific channel blockers, such as the trivalent cations La^3+^ and Gd^3+^ [12,55,56], or commonly used nonselective inhibitors of receptor-mediated Ca^2+^ entry, verapamil or SKF96365 [55]. Due to their wide range of targets, these blockers were only of limited use for the characterization of TRPC3 in native tissues and not suitable for the development of therapeutic application. A first step toward the more specific targeting of TRPC3 function was achieved by He et al., showing that the 3,5-bis(trifluoromethyl)pyrazole (YM-58483 or BTP2), which was initially described as an inhibitor of T-lymphocytes store-operated Ca^2+^ entry (SOCE) [57,58], was inhibiting TRPC3 channel activity in different cell types including DT40 B-lymphocytes [59]. Based on the observation of BTP2 inhibition of TRPC3 conductance activated by carbachol (CCh) or oleoyl-acetyl glycerol (OAG; Figure 1) [59], these authors clearly identified the pyrazole derivative as an inhibitor of TRPC3. Since BTP2 still lacked appreciable selectivity among different Ca^2+^ entry pathways, Kiyonaka et al. synthesized and characterized a series of pyrazole derivatives to discriminate between SOCE, TRPC,3 and other TRPC isoforms. These authors reported a new pyrazole 3 (Pyr3) inhibitor of TRPC channels (Figure 1) with a striking preference for TRPC3. Notably, 3 µM of Pyr3, which effectively inhibited TRPC3, failed to suppress TRPC6, TRPM2, TRPM4, and TRPM7 channels overexpressed in HEK293 cells. Pyr3 was suggested to inhibit TRPC3 channels from the extracellular side and photoaffinity labeling of Pyr3 showed a strong incorporation of the inhibitor into TRPC3 but not into TRPC6 channels [60]. The exact site of molecular interaction has not been clearly defined, and even a principle blocking mechanism by occluding the permeation pathway has not been conclusively delineated. Pyr3 certainly advanced the field by enabling a pharmacological dissection of closely related TRPC channel subtypes. However, later investigations on the selectivity of pyrazole inhibitors demonstrated that Pyr3 inhibits STIM/Orai Ca^2+^ entry complexes [61]. The latter authors identified other pyrazole derivatives that are indeed able to discriminate between TRPC3 and Orai-mediated SOCE, including an acceptably selective TRPC3 blocker (Pyr10). This pyrazole blocked recombinant, homomeric TRPC3 channels were highly potent (IC_50_ > 0.72 μM) but affected SOCE only at concentrations more than one order of magnitude higher (IC_50_ > 10 μM) [61].

Another screening study by Washburn et al. identified two potent and selective thiazole inhibitors of TRPC channels. These compounds, assigned GSK2332255B (GSK255B) and GSK2833503A (GSK503A), are anilino-thiazoles and feature a nanomolar potency for blocking TRPC6 and TRPC3 and reportedly lack significant effects on many other calcium-permeable channels [62]. Since TRPC3/6 channels were implicated in the pathogenesis of hypertension and hypertrophy, GSK255B and GSK503A were tested in animal models of cardiac hypertrophy and remodeling. GSK255B and GSK503A, most likely by a combined suppression of TRPC3 and TRPC6 conductance, reduced hypertrophy and fibrosis induced by pressure overload in rodents [63].

Other TRPC3 blocking agents with ill-defined selectivity have been introduced, such as norgestamate [64], HC-C3A [40], 4-({(*1R*,*2R*)-2-[(*3R*)-3-aminopiperidin-1-yl]-2,3-dihydro-1*H*-inden-1-yl}oxy)-3- chlorobenzonitrile (SAR7334) [65], and 2-(benzo[d][1,3]dioxol-5-ylamino)thiazol-4-yl)((*3S*,*5R*)-3,5-dimethylpiperidin-1-yl)methanone (BTDM) [8]. The latter compound (BTDM), albeit incompletely characterized at the cellular and tissue level, was able to delineate TRPC6 and TRPC3 structure by cryo-EM [8]. Consequently, a BTDM binding site was localized within the TRPC6 tetrameric complex (Figure 1). Importantly, another study resolving the TRPC3 structure by cryo-EM [7] identified a highly charged extracellular cavity with close structural relation to the pore domain, therefore representing a potential interaction site for inhibitors and/or modulators of the channel. Thus, the first high-resolution structural information on the drug binding site in the TRPC3 complex has emerged, and this information will promote the development of therapeutic targeting of TRPC3 (Figure 1).

## 4. Endogenous and Synthetic Channel Activators

A hallmark of the cellular regulation of mammalian TRPC channels is the intimate linkage between channel activity and membrane lipid composition. TRPC3 is, for a large part, governed by its membrane lipid environment. Not only production of diacylglycerols (DAGs; Figure 1) in response to phospholipase C (PLC) activation activates the channel, but also phosphatidylinositol 4,5-bisphosphate PIP_2_ as a precursor of DAG formation, has been identified as a determinant of TRPC3 activity [66]. Both PIP_2_ and DAG appear to promote channel activity. Synthetic and photoswitchable DAGs have been introduced as activators that enable optical control of TRPC3 activity. As a highly active, unnatural lipid activator, a DAG with two arachidonyl-mimicking azobenzene moieties was introduced (OptoDArG; Figure 1) [14]. In addition to glycerol derivatives, membrane cholesterol was shown to initiate and enhance TRPC3 activity [67]. The effect of cholesterol was attributed in part by enhanced recruitment of the channel into the plasma membrane. Importantly, two distinct regulation mechanisms were reported for TRPC channels including TRPC3 in particular. This is, on the one hand, an increase in the open probability in membrane resident channels, as shown at the level of single TRPC3 channels for PLC-mediated activation, which is characterized by destabilized closed channel conformations [14]. On the other hand, the recruitment of a vesicular pool of TRPC channels into the plasma membrane was proposed. Through this mechanism, certain activating stimuli might enhance channel availability, and thereby TRPC3 currents and downstream signaling [68]. In this respect, TRPC3, in contrast to its close relative TRPC6, displays constitutive activity in resting cells, at essentially low PLC activity. Suppressed basal activity in TRPC6 was found to be related to the channel’s glycosylation pattern. Dual glycosylation at two asparagine residues was found to be crucial for maintaining the basal activity of TRPC6 low compared to monoglycosylated TRPC3, which is marginally permeable for ions in a resting state [69]. Constitutive gating activity appears largely independent of basal levels of DAGs in the membrane, as a DAG-insensitive mutant (G652A) of TRPC3 retained constitutive activity [14]. The same mutation also retained sensitivity to activation by a synthetic activator GSK1702934A (GSK; Figure 1) that clearly acts in manner different from DAGs to enhance the open probability of TRPC3 [14]. Xu et al. introduced this small and apparently selective agonist of ligand-gated TRPC channels, which activated TRPC3/6 overexpressed in HEK293 cells and increased the perfusion pressure of isolated rat heart and transiently increased blood pressure in conscious Sprague Dawley rats [70]. Later, Qu et al. introduced a series of TRPC-selective agonists, which lacked effects on other members of the TRP family (TRPA, TRPM, and TRPV). These agonists were pyrazolopyrimidine-based and remarkably potent (EC_50_ in the nanomolar range) in the activation of recombinant TRPC3, TRPC6, and TRPC7 channels [71]. These synthetic small molecule agonists of TRPC channels (GSK-related and pyrazolopyrimidine-based structures) appear to bypass the PLC pathway and the TRPC lipid-gating machinery. Importantly, GSK has been found to exert little to no effect on membrane conductance of cardiomyocytes at an essentially low level of TRPC3 expression in the murine heart, but induced TRPC3 currents when the channel was overexpressed in a genetic mouse model [22]. This indicates a relatively high specificity of the GSK activator for TRPC3/6/7 channels, since none of the abundant voltage-gated cardiac conductances were affected [22]. For pyrazolopyrimidine-based agonists, Qu et al. confirmed the selectivity and efficiency in stimulating endogenous TRPC3/6 activity in rat primary glomerular mesangial cells by Ca^2+^ measurements [71]. Of note, limited therapeutic interest has been expressed as of yet for synthetic activators of TRPC3, since most TRPC3-associated pathologies are related to either an enhanced expression or a gain in function of phenotypes. Hence, drug development activities have focused primarily on selective antagonists or blockers of the channel. Nonetheless, TRPC3 has been identified as promoting proliferative cell phenotypes [16,29,36] and may be involved not only in maladaptive tissue remodeling but also in tissue regeneration. Therefore, unconventional approaches that provide high spatial precision of intervention, such as photopharmacology, may create the possibility of a therapeutic application for TRPC3 activators.

## 5. New Insights into the Ligand Binding Domains in TRPC3

**Figure 2:**
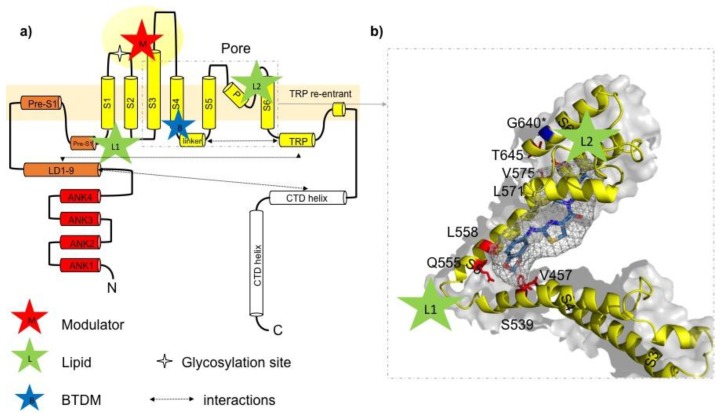
Ligand-channel interactions and potential drug binding sites in TRPC3. (**a**) Schematic illustration of the domain structure of one TRPC3 channel subunit according to information provided in Fan et al. [7]. Lipid binding sites (green stars) are indicated with L1 (formed by LD9, pre-S1, S1, S4, and S4–S5 linker) and L2 (between p-loop and S6 helix); potential modulator binding site (M) represented by a cavity (extracellular domain) formed by the extended S3 helix, S1–S2 and S3–S4 linkers as previously identified [7]. Proposed BTDM binding site formed by S3, S4–S5 linker, S4, S5, and S6 identified by Tang et al. [8]. (**b**) Detailed view on postulated 2-(benzo[d][1,3]dioxol-5-ylamino)thiazol-4-yl) ((*3S*,*5R*)-3,5-dimethylpiperidin-1-yl)methanone (BTDM) binding site in TRPC3: amino acids in the TRPC3 sequence, corresponding to the TRPC6 BTDM binding site are marked in red. The BTDM molecule is only schematically introduced into the TRPC structure and not adjusted in size. The glycine residue G652 (here G640, isoform 1/Q13507-1 in UniProt) identified as crucial for recognition and accommodation of lipid activators is highlighted in blue [14]. The BTDM molecule is schematically placed into the proposed binding site.

The delineation of TRPC3 and TRPC6 structures by cryo-EM microscopy approaches succeeded in the localization of potential binding sites for blockers, modulators, as well as endogenous lipids. Tang et al. presented the structure of homotetrameric TRPC3 complexes at 4.4 Å [7]. A resolution along with the structure of TRPC6 (3.8 Å resolution), in which binding of the high affinity inhibitor BTDM was localized between the S1–S4 voltage sensor-like domain (VSLD) and the pore domain. This is a position in which an interaction is likely to hinder gating movements (Figure 1). The BTDM binding site is conserved between TRPC6 and TRPC3, and interaction of this potent inhibitor structure with the channel appears not to overlap with lipid regulation, as some mutations that prevent BTDM binding did not interfere with activation by DAGs. Tang et al. performed a structure analysis of TRPC3 reconstituted into nanodiscs in the presence of the diacylglycerol activator OAG, but obtained a closed channel conformation and could not discern the presence of the lipid activator [8]. Fan et al. reported the structure of tetrameric TRPC3 complexes in a lipid-occupied closed state at a 3.3 Å resolution and localized two lipid interaction sites without identifying the molecular nature of the lipid species [7]. One lipid molecule occupied a position between a pre-S1 elbow-like structure and the S4–S5 linker representing a pivotal element of TRPC gating. A second lipid-like density was found within a lateral fenestration in the pore domain (Figure 2). This second and potentially lipid-interaction site is close to the previously recognized critical LFW motif in the pore domain. It was identified by our laboratory as a structure essential for DAG recognition and lipid gating in the channel using homology modeling combined with structure-guided mutagenesis and a novel optical lipid-clamp approach [14]. Observation of a closed channel state may reflect either a desensitized or inactivated state of the channel or the presence of an inert, non-activating lipid species that occupies the channel in its resting state.

Both natural as well as synthetic modulators are likely to occupy distinct binding pockets to interfere with the gating machinery involving conformational changes in both the S1–S4 VSLD and the pore domains. Fan et al. identified a charged extracellular cavity formed by an extended S3 helix and the S3–S4 linker. This extracellular domain connects to the pore domain via hydrophobic interactions and represents a potential extracellular modulatory site for small molecules including pyrazole inhibitors [7].

Importantly, these advances in TRPC3 structural biology have created a basis for further advances with TRPC3 synthetic biology in terms of optogenetics and photopharmacology as outlined below. Availability of structural information at the atomic level will allow for tethered ligand approaches and optogating by crosslinking domains recognized as gating elements.

## 6. TRPC3 Photopharmacology—A Therapeutic Perspective

Therapeutic targeting of a multifunctional signaling molecule that is expressed in a wide range of tissues, as is the case for TRPC3 [23], typically requires refined pharmacological strategies to obtain sufficient tissue and/or cell-type specificity for clinically useful interventions. Of note, information on the viability in a genetic mouse model, which lacks expression of all TRPC species [72], indicated that the nonselective and simultaneous block of multiple TRPC conductances might not necessarily generate severe side effects in healthy tissues and organs. It is tempting to speculate that the impact of TRPC3 in organ dysfunction is based on either a certain expression profile of TRPC genes and/or a certain cell- or phenotype-dependent signaling signature of TRPC3, along with its closer relatives in diseased states. Hence, gaining understanding on the cell-type specific function of these channels in normal and pathological states is important. Cell-type specific interventions might be achievable by manipulating TRPC3 signaling in a spatiotemporally precise manner using photopharmacology.

Initial attempts adopted the caged ligand concept to gain optical control over TRPC channels [73]. This approach was based on the availability of caged lipid activators of TRPC3/6 channels. DAGs were fused to coumarin and nitroveratroyl molecules to prevent immediate interaction with their biological targets [74]. A significant increase in Ca^2+^ was generated in HeLa cells, which endogenously express TRPC3 and TRPC6 channels upon photorelease of the active lipid mediator with ultraviolet (UV)-A light. Notably, caged stearyl-arachinonyl-glycerol (SAG) was found to be the most potent agonist amongst different DAGs, consistent with the initial characterization of lipid sensitivity by Hofmann et al. [5]. Photoreleased SAG triggered the most sustained and largest increase in Ca^2+^ concentrations compared with other lipid activators, such as stearyl-linoenyl- and 1,2-dioctanoyl-glycerol. NiCl_2_ and TRPC non-selective blocker SKF-96365 almost completely suppressed response in HeLa cells to lipid uncaging, suggesting a TRPC-dependent Ca^2+^ influx [74].

Disadvantages of the caged-lipid approach include the principle irreversibility of the chemical switch and the unavoidable generation of a second molecular structure represented by the released cage. Tiapko et al. clearly showed the undesirable off-target effect with the caged lipid strategy. Photorelease of caged 1,2-DOG was found to generate an artifact caused by UV light application in the presence of the generated free coumarin moiety, whereas the UV light itself did not affect membrane conductance. Hence, off-target effects, due to the phototoxicity of the caging structure, requires consideration and is expected to limit the therapeutic value of caged ligand strategies [75].

As an alternative approach, the photochromic ligand approach has been successfully adopted for TRPC photopharmacology [14,76]. Herein, the structure and activity of the ligand was reversibly controlled by light. As a conformation-flexible, light-sensing structure, azobenzene was introduced into the DAG structure. The biological activity of these photochromic activators is controlled by *cis-trans* photoisomerization of the azobenzene, which resides within the aliphatic side chains of the DAG. Azobenzene-modified DAGs successfully served as tools for studying TRPC cell-specific functions as well as ligand-protein interactions and channel activation-deactivation kinetics [14,76,77,78].

Initial characterization of different photochromic DAGs demonstrated precise and reversible control of Ca^2+^ signals in HeLa cells, with probes designated as PhoDAGs. PhoDAG-1 resembles SAG with the arachidonyl side chain replaced by a corresponding structural element containing the azobenzene moiety. Two other structurally related lipids were predicted to functionally mimic 1,2-DOG (PhoDAG-2 and PhoDAG-3). Typically, the *trans*-conformation exerted little or no effect on intracellular Ca^2+^, whereas *cis*-adopted molecules efficiently triggering Ca^2+^ influx through the plasma membrane. These actions were fully reversible and allowed for precise cyclic control of the signaling function [78].

With these new tools, Leinders-Zufall et al. demonstrated manipulation of mTRPC2 channels, endogenously expressed in neurons of murine sensory neurons from main olfactory and vomeronasal epithelium. As localization of these channels is confined to certain cell types and cellular structures, the spatial precision of the new technology provided important insight into DAG-sensitive TRPC2 function in the mouse olfactory system [76]. In another study, a photochromic DAG, containing two arachidonic acid-mimicking photochromic moieties, designated as OptoDArG, was introduced and successfully used to investigate the lipid sensing machinery [14]. This study used the intriguing temporal precision of the method and uncovered not only a structural element involved in lipid recognition by TRPC3, but also a cooperative slow gating processes, residing in a subunit interface within the pore domain. Such cooperative gating processes are potentially important determinants of frequency dependence of signaling, thereby generating specific signaling signatures dependent on complex composition, cellular localization of the complexes, or upstream signaling patterns. Detailed elucidation of cell- and phenotype-specific TRPC3 signaling features appears to be an essential next step toward the therapeutic targeting of this molecule. 

## 7. Conclusions

Emerging technologies for precise spatiotemporal manipulation of the activity of TRPC channels by light, along with an increase in available structural information on drug interaction sites and gating processes in TRPC channels, are expected to promote the development of novel therapeutic concepts. TRPC photopharmacology will advance the field by enabling exact control over gating pattern, the option of spatially precise manipulation, as well as by providing a basis for efficient all-optical drug screening. As such, cell-type and tissue-specific targeting of TRPC3 and respective interventions of therapeutic value appear feasible and are awaited.
